# Supplementary material for: The Rapid Activation of MYDGF Is Critical for Cell Survival in the Acute Phase of Retinal Regeneration in Fish
Source: Int J Mol Sci. 2025 Jul 27;26(15):7251. doi: 10.3390/ijms26157251 (PMC12346886; doi:10.3390/ijms26157251)
Supplement: Supplementary file 1 [file ijms-26-07251-s001.zip › ijms-3702271-Supplementary Materials.pdf]

Suppl. Figure S1

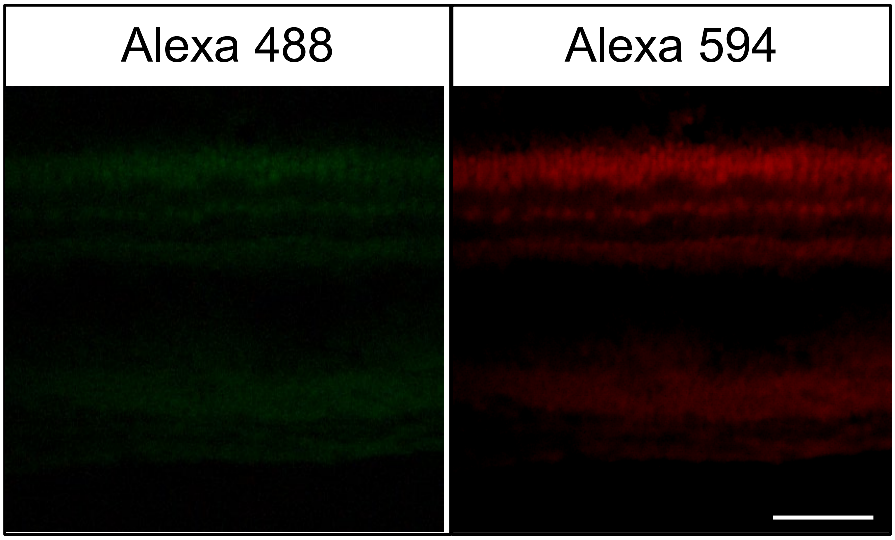

(Scale, 50μm)

Suppl. Figure S1. Negative control for fluorescent staining using retinal sections 1 h following optic nerve injury. Fluorescent staining was performed without the use of a primary antibody. Minimal nonspecific fluorescence was observed with Alexa Fluor 488 (green), whereas Alexa Fluor 594 produced slightly stronger nonspecific fluorescence, particularly in the outer nuclear layer (ONL).

Suppl. Figure S2

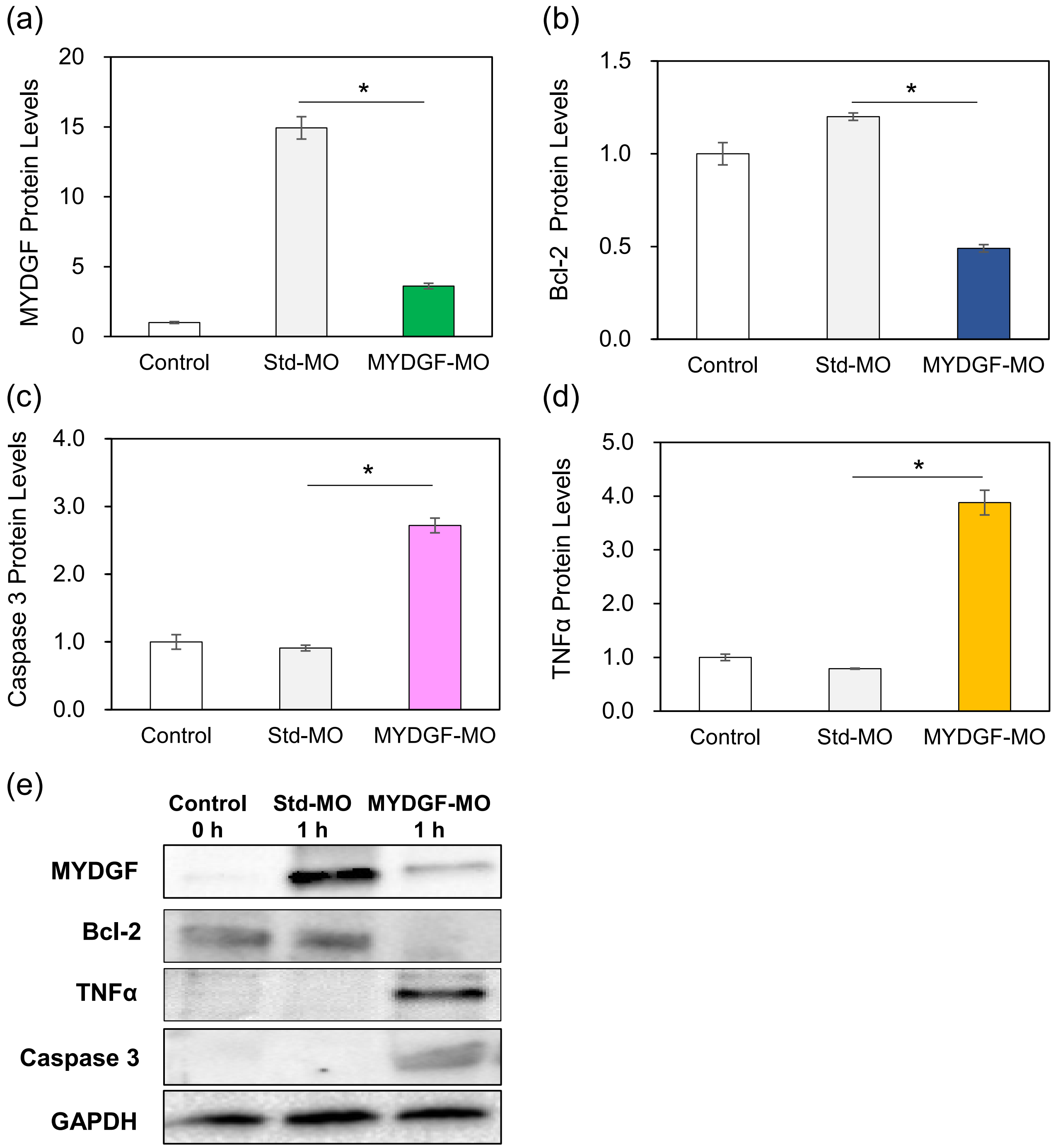

Suppl. Figure S2. Western blot analysis of the effect of MYDGF-specific Morpholino (MYDGF-MO) in the zebrafish retina 1 h after ONI. (a) Intraocular injections of MYDGF-MO 20 h before ONI completely suppressed MYDGF protein expression compared to the control and the standard-MO group (Std-MO). (b) Similarly, the anti-apoptotic protein Bcl-2 was markedly suppressed in the MYDGF-MO injection group (MYDGF-MO). (c) In contrast, MYDGF-MO significantly activated the apoptosis-inducing factor caspase-3 and (d) the inflammatory factor TNFα. (e) Representative examples of western blotting results for each factor are shown. *$p < 0.05$ vs. Std-MO. $n = 3$. Each experiments was repeated three times with consistent results.

Suppl. Figure S3

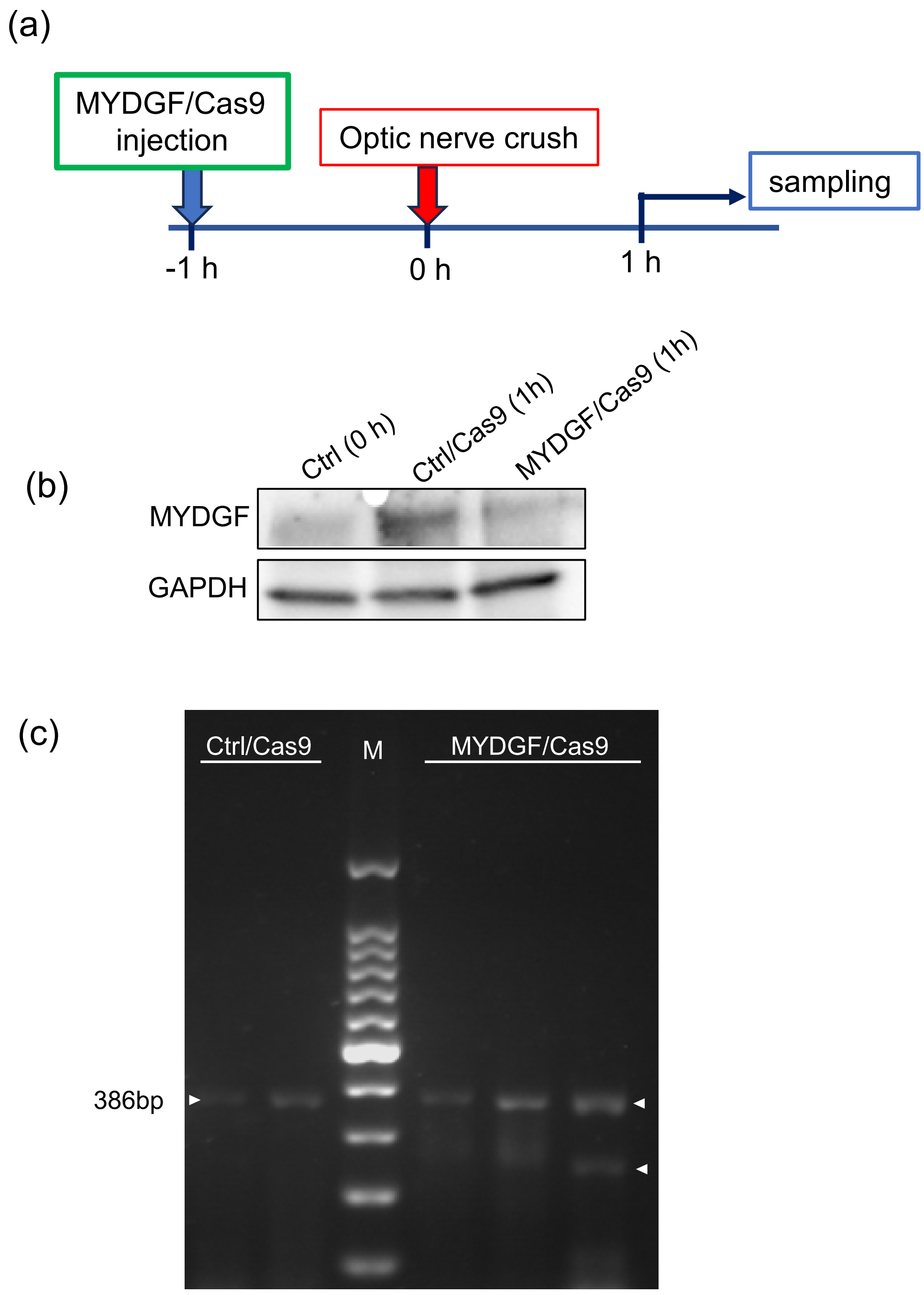

Suppl. Figure S3. Effect of MYDGF knockdown with the CRISPR/Cas9 system using the intravitreal injection method. (a) CRISPR/Cas9 System treatment and sampling schedule. (b) Western blot analysis using retinal samples collected 1 hour after optic nerve crush revealed that MYDGF protein expression was elevated in the control/Cas9 group (Ctrl/Cas9) compared to the untreated samples (Ctrl 0 h). In contrast, the MYDGF/Cas9-treated group showed a marked suppression of MYDGF protein expression. GAPDH was used as a loading control. (c) Indel detection analysis was performed to evaluate the knockout efficiency of the Cas9 system. In the control group, genomic DNA was successfully amplified by PCR, showing a single clear band. In contrast, the MYDGF/Cas9-KO group exhibited two distinct bands, indicating the presence of non-specific cleavage and suggesting that indel mutations were introduced at the target site.

Suppl. Figure S4

(a)

```
>zf-mydgf
cagaactgccccatcctatgaagaacatacaggaacagtacaggcacctttttcattcactacataaacgcagatct
cctgtctgtaaagcatcatggcatttattgtgcacatgaaatggtttgtgaatcttctgctgctgtttgttgtgctt
tgtgaactgtgttctgctgaaaggaccaaaacactggacttcgatgtcaaacctggaggagttgtgcagactttctc
tgcaaaacttaagaagtataaatgcaccttcacatatgcatgccaaggaggaaccaatgagcaatggcaaatgagtg
tcggactaagtgacgatgagcaaatgtttcctgttcagtatggaggccccaagggaagtcctacttgtttttacg
cagttcaaagccgagataaaggagccaagatcgagtacgccaccgcatattcccagacggccgtgggtggacagag
ggatgttgctttgaaagaagaagagtacatagtgtcagagtctgcagtgacacaaagagatggaaaattccattcgg
agctttctaagctcactgtcattggtcgaatacggcatgatgaactctgattggccgattcgaaggacgttggttat
ttaacagctttggacacaattatctgctcacggtcacaagtcaagcgaaggaaatcacagcactggacgttctgaaa
aatgaatttactgtgatagaaaagatttcttacagatgccagtggccaaaggttggagtgatcatcttagagaggta
gacgaaaagtctgttattggatattataaggggtaaaacaaattgtacatgcacatttttttcatatttgtaaggtg
ttatgaaggcttctttatattcagtgcagaacagcagattgatttctgttcttgtcagaatttatgggttttctagg
gttacagtctgactttgattcatacctgcaaagctgattatcaagagtgctcatacttactttcttaggctggtgta
aaggcacgcttgtagacaattcccactaaatctttggggtgtgtcagaatcctttgagtatttctgtggtggaggaa
aaaacatcaagatggttactacagttccttttgtctcccagtcatgctttattctgataaccgatgtatcggaattt
gaataaatctgttatcagaacaatttgacttctggccttactatttgctgactttgtatacctaatattttgcgta
aagtaagtgaattgtgtctgtttgaaagcaacaacctctgagggctgttttttttgcactcttaagggtgcaaaatg
tgtgtaattctgctgcataaaattaaaacaagctacaaatttgctgattctgttcaagatttgtccagtcagctctt
tgaaaggcttgtcggtatcccaactgtgaataaaagagcctgcaatgcaattcacaagctattaaagaaattaatgt
cttgagaaccaaaa
```

sgRNA1 □ (exon 1) , sgRNA2□ (exon 2), sgRNA3□ (exon 6), sgRNA4 □ (exon 6)

(b)

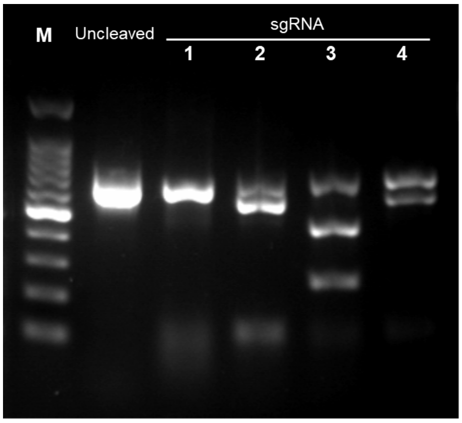

(c)

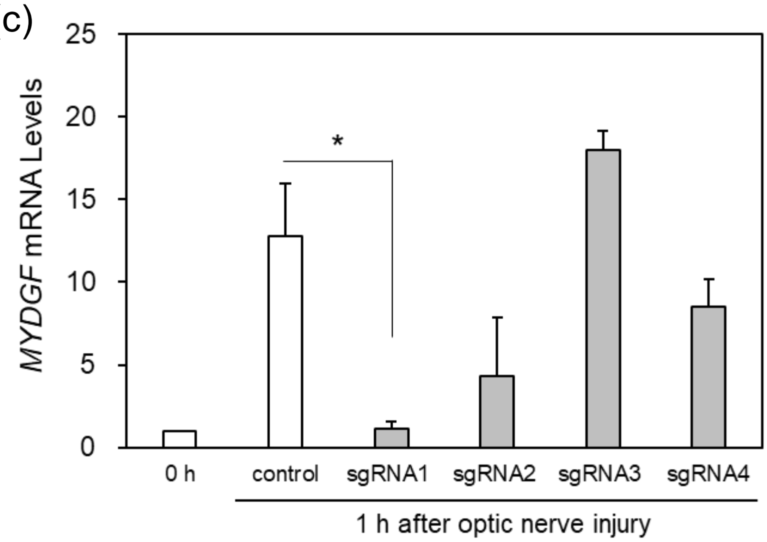

Suppl. Figure S4.

Four sgRNA designs and their knockdown effects on *MYDGF* in vitro and in vivo. (a) Guide RNAs were designed by CRISPRdirect, a free online design tool, along with four single guide RNAs (sgRNAs). The PAM sequence (5'-NGG-3') of the *MYDGF* gene was searched and the 20 bases upstream of this sequence were designated as sgRNA candidates (sgRNA1 to sgRNA4); this sequence was colored with markers. (b) Guide RNA cleavage activity of the *MYDGF* gene was first confirmed in an *in vitro* experimental system. All sgRNAs showed cleavage activity of the target PCR products (uncleaved). (c) A total of 0.5 μL/eye of a mixture of target-specific sgRNA (50 ng/μL) and recombinant Cas9 nucleases (500 ng/μL) was directly injected into zebrafish eyes. The mRNA expression of *MYDGF* was then measured in samples 1 hour after optic nerve injury. Only sgRNA1 was observed to significantly suppress *MYDGF* mRNA expression.

Suppl. Figure S5

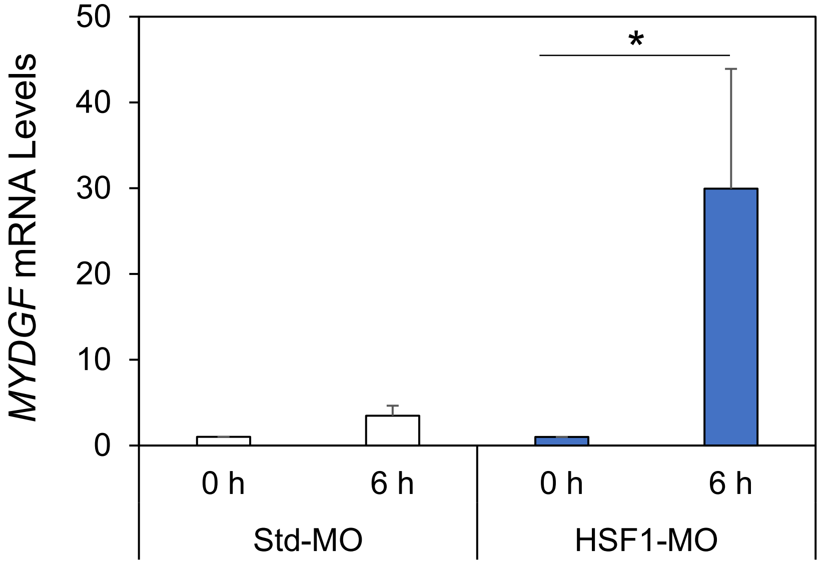

Suppl. Figure S5.

Treatment with HSF1-MO markedly increased MYDGF mRNA 6 h after optic nerve injury. Statistical analysis was performed using one-way ANOVAs, followed by Scheffe's multiple-comparison tests. Data are expressed as the means ± SEMs, with statistical significance set at $*p < 0.05$. n=3.
